# Metatypical Basal Cell Carcinoma with Intravascular Invasion

**DOI:** 10.7759/cureus.3401

**Published:** 2018-10-02

**Authors:** Shawn Shih, Christina Dai, Ahmed Ansari, Jeffrey Greenwald

**Affiliations:** 1 Internal Medicine, University of Central Florida College of Medicine, Orlando, USA; 2 Dermatology, University of Central Florida College of Medicine, Orlando, USA

**Keywords:** basal cell carcinoma, metatypical basal cell carcinoma, bcc, basosquamous

## Abstract

Basal cell carcinoma is the most common malignancy worldwide, but it very rarely metastasizes. Perineural invasion in basal cell carcinoma has been well documented in the literature, but evidence of intravascular invasion has rarely been reported. We describe a rare case of metatypical basal cell carcinoma with intravascular invasion and discuss the clinical management associated with this presentation. The patient was successfully treated with two stages of Mohs micrographic surgery.

## Introduction

Basal cell carcinoma (BCC) is the most common malignancy in humans worldwide [1]. Although it makes up three quarters of all reported skin cancers, BCC rarely metastasizes, with the incidence reported to range from 0.0028% to 0.5% [1]. Metastasis is thought to occur via lymphatic and hematogenous spread, most commonly to regional lymph nodes, followed by the lung and bone. While perineural invasion (PNI) in BCC and squamous cell carcinoma (SCC) has been well documented, histologic evidence of intravascular invasion has rarely been reported. Here we present a rare case of metatypical basal cell carcinoma with intravascular invasion located on the lateral shoulder, successfully treated with two stages of Mohs micrographic surgery (MMS).

## Case presentation

**Figure 1:**
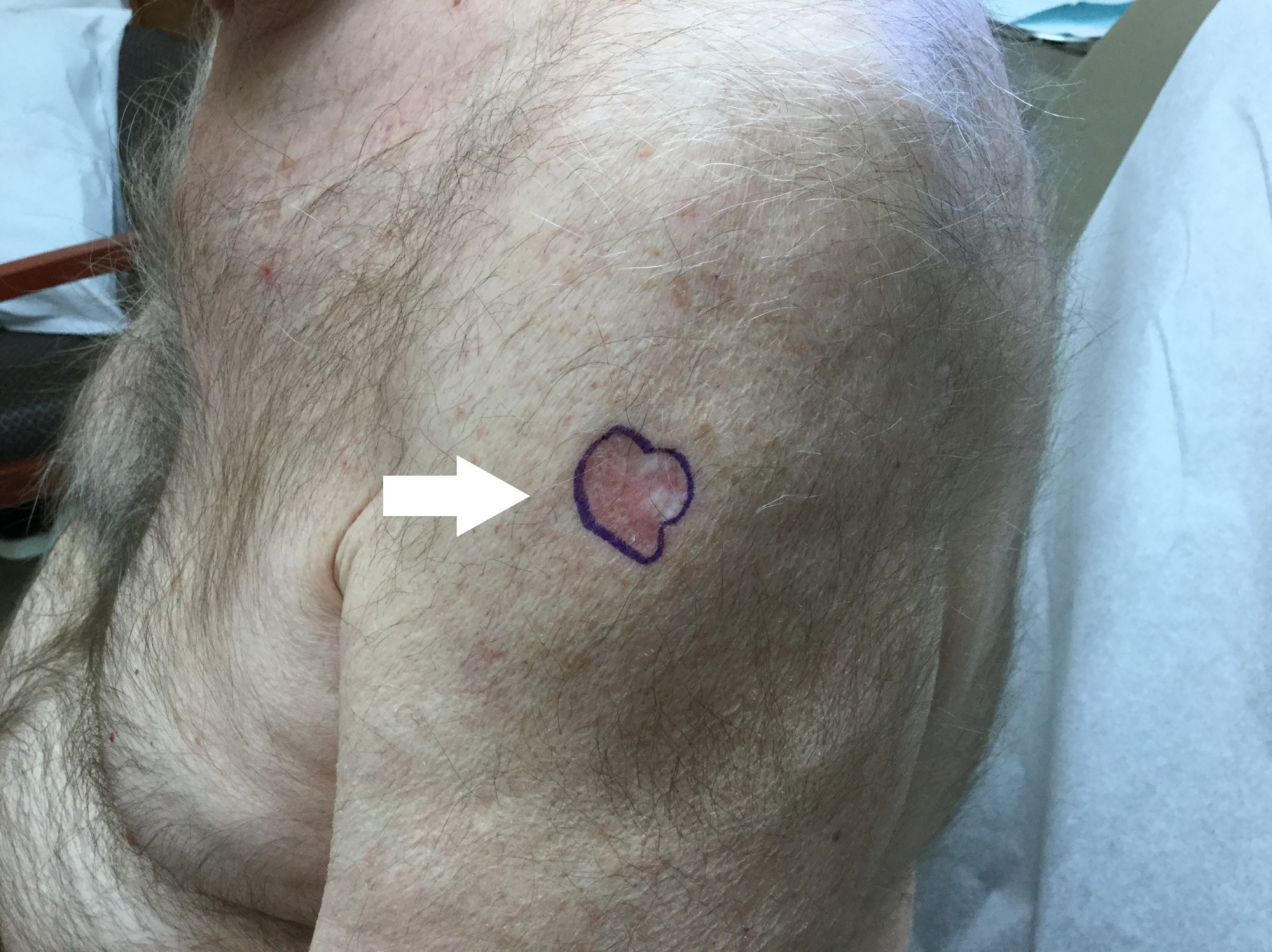
Metatypical basal cell carcinoma located on the left lateral shoulder. Basal cell carcinoma presenting as a 2.8 cm psoriasiform patch located on the left lateral shoulder at a site previously treated for basal cell carcinoma.

**Figure 2:**
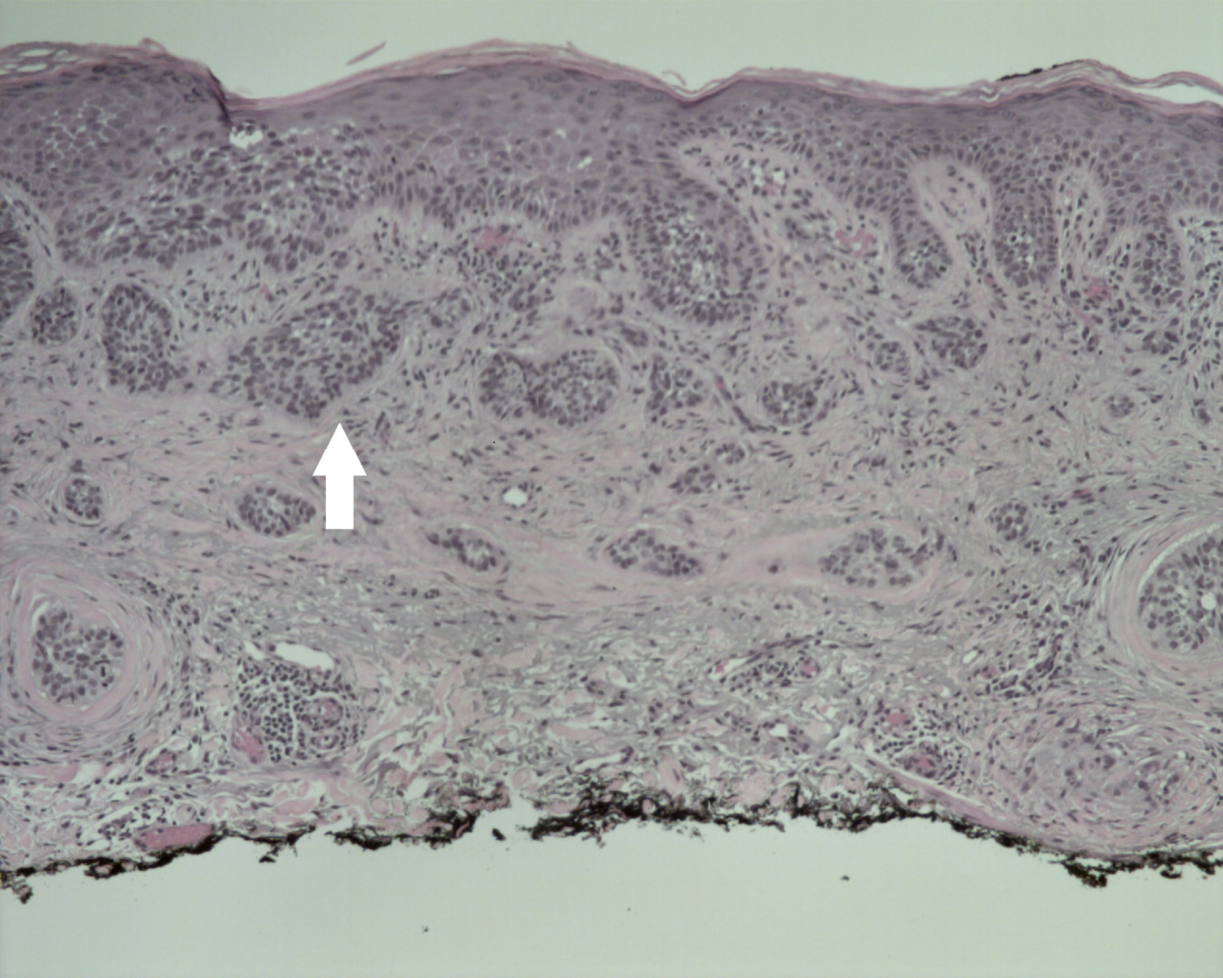
Histology of metatypical basal cell carcinoma. Basaloid nests with tumor-stromal clefts and overlying squamoid differentiation of nests beneath an inflamed epidermis. (hematoxylin-eosin, original magnification ×10)

**Figure 3:**
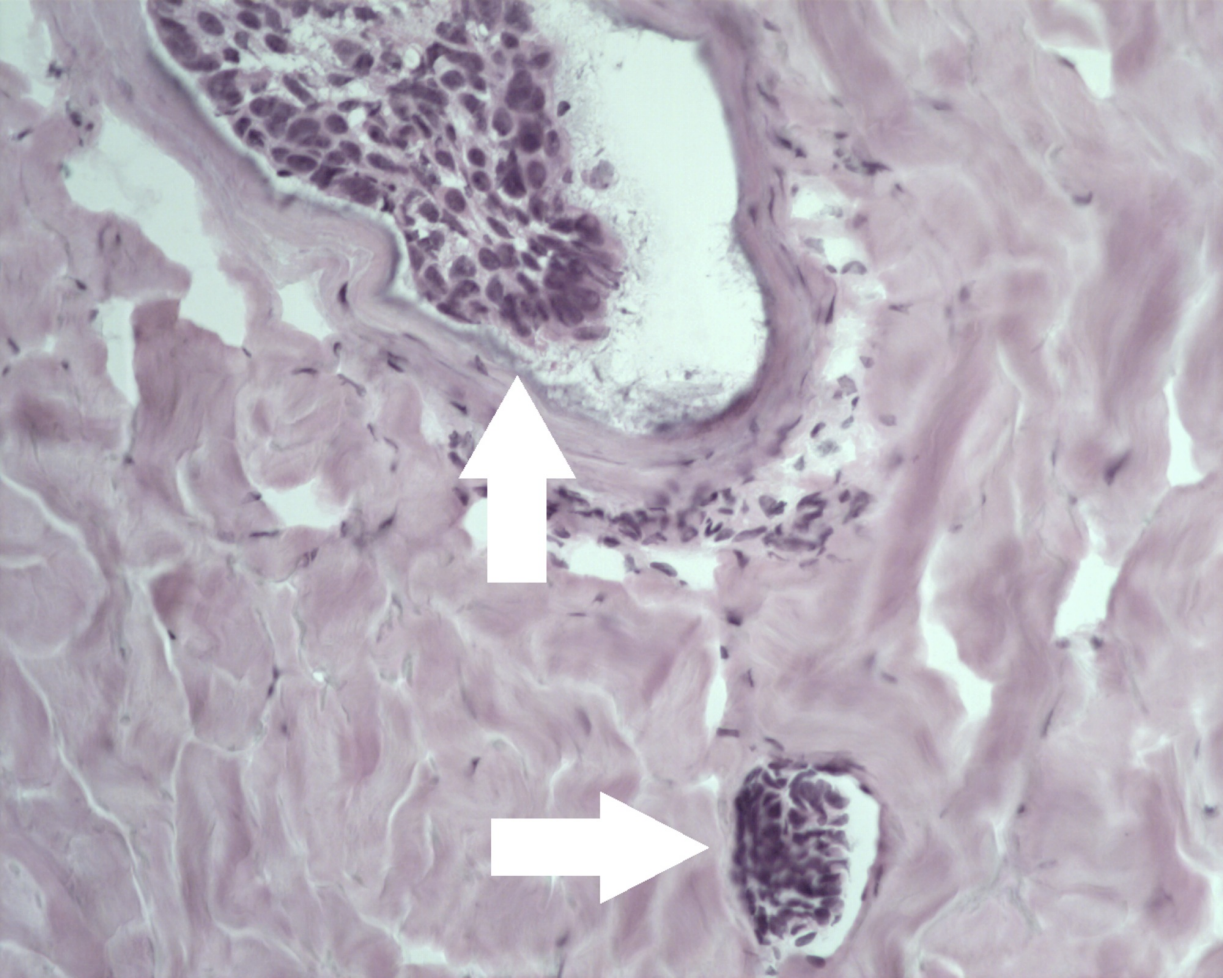
Metatypical basal cell carcinoma with intravascular invasion. Basal cell carcinoma seen in the intralumenal space of a small vessel (top arrow) and another smaller (bottom arrow) on stage 1 of Mohs micrographic surgery. (hematoxylin-eosin, original magnification ×20)

A 76-year-old Caucasian male with a history of SCCs, BCCs, and previously treated metastatic melanoma presented to the dermatology clinic in October 2017 with an erythematous lesion of two-month duration on the left lateral shoulder. He had a history of melanoma in situ of the abdomen excised in 2003, lentigo maligna melanoma of the scalp excised in 2005, and metastatic melanoma of the scalp in 2007, treated with interferon for a year. Physical examination of the left upper extremity revealed a psoriasiform patch 2.1 cm in diameter on the left lateral shoulder (Figure 1). The lesion was located at a site previously treated for BCC via shave biopsy and destruction six months prior. Due to high suspicion for recurrence of a previously treated BCC, the new lesion was biopsied via shave method. Histologic examination revealed basaloid nests with tumor-stromal clefts and overlying squamoid differentiation of nests beneath an inflamed epidermis (Figure 2), and diagnosis of metatypical basal cell carcinoma was established. MMS was recommended as the treatment of choice due to the tumor’s large size (2.8 x 2.1 cm), recurrence after prior destruction, and metatypical histology. The patient returned in December, 2017 for MMS, and a tumor-free plane was reached after two stages. However, intravascular involvement was noted on stage one of the Mohs sections (Figure 3), and a second stage revealed negative surgical margins. There was no perineural involvement. The patient was then referred to an oncologist for further studies with positron emission tomography (PET) and computed tomography scans, which revealed no metastatic disease. Complete metabolic panel and complete blood count were also within normal limits. Follow-up visit two weeks post-op revealed a clean wound. The patient elected to follow up at the dermatology clinic only. To date, no systemic signs or symptoms were noted.

## Discussion

This is a rare case of metatypical basal cell carcinoma and intravascular involvement. A comprehensive literature search found seven previously reported cases of BCC with intravascular invasion of other subtypes (Table 1) [2-7].

**Table 1:** A summary of cases found in the literature of basal cell carcinoma with intravascular involvement. The cases are arranged numerically in order of publication date, starting from the most recent. Patient 1 through 5 had intravascular involvement of the primary tumor. Patients 6 and 7 had intravascular involvement of a recurring tumor. Patients 5, 6, and 7 had multiple recurrences and subsequent metastases. MMS: Mohs micrographic surgery

Case No.	Age at presentation	Sex	Primary tumor site	Histological subtype	Treatment	Outcome
1	81	Female	Nasal tip	Micronodular and sclerosing	Surgical excision and adjuvant radiation	No recurrence at four months
2	75	Male	Left nasal sidewall	Nodular and morpheaform	MMS (three stages)	Follow-up not reported
3	96	Female	Posterior helix	Not reported	MMS (two stages)	No further workup
4	51	Male	Upper chest	Infiltrating and micronodular	Surgical excision	Follow-up not reported
5	51	Male	Right posterior upper shoulder	Infiltrating	MMS after two recurrences were treated with electrodesiccation and curettage and surgical excision, respectively	Death from pulmonary metastasis 13 years later
6	71-72	Male	Left chin	Not reported	Not specified	No recurrence at nine years
7	27	Male	Left cheek	Infiltrating	Surgical excision	Death from pulmonary metastasis four years later

Since metastatic BCC is associated with a five-year survival rate of only 10%, it is important to determine whether BCC with intravascular involvement is associated with an increased risk of metastasis and thus warrants more aggressive workup and treatment [8]. More aggressive histologic subtypes of BCC, including morpheaform, micronodular, and metatypical, are more likely to metastasize. Other risk factors for a metastatic event include the male sex, tumor of the head and neck, large tumor size (>2 cm), recurrence at the primary site, immunosuppression, and PNI. PNI in non-melanoma skin cancers is associated with a much higher risk of local recurrence and metastasis, despite a much higher risk in SCC than in BCC [9]. While there exist National Comprehensive Cancer Network guidelines for management of non-melanoma skin cancers with PNI, the clinical relevance and therapeutic implication of intravascular invasion in BCC is not well understood due to its rarity. Intravascular involvement was thought to be associated with an increased risk of hematogenous spread and consequent metastasis, however, there is a paucity of literature to support.

PET scan and sentinel node biopsy (SLNB) are often used to detect metastatic disease in various cancers. Although their utility for BCC is not well documented due to low incidence of metastasis, PET scan and SLNB have been successful in detecting metastatic BCC according to some reports [10-11]. Therefore, PET scan and/or SLNB are reasonable measures to undertake in a patient with BCC when multiple risk factors for metastasis are present. Our patient had numerous risk factors, including large tumor size, recurrence, the male sex, and metatypical subtype. Our case, along with the rare previous reports, raises the important question of the potential clinical significance of finding intravascular BCC and the extent of workup indicated as well as the likely prognosis. In this case, with negative PET scan findings and no clinically palpable lymph nodes, the patient and his oncologist elected not to have a sentinel node biopsy performed. However, frequent close follow-up at the dermatology clinic is warranted due to his previous history of metastatic melanoma as well as the poorly understood prognosis of BCC with intravascular invasion.

## Conclusions

We describe a rare case of metatypical basal cell carcinoma with intravascular invasion and discuss the clinical management associated with this presentation. The patient was successfully treated with two stages of Mohs micrographic surgery. While the clinical significance of intravascular invasion remains unknown, our patient had multiple risk factors for metastasis, including large tumor size, recurrence, the male sex, and metatypical subtype, but PET scan findings were negative. However, frequent close follow-up at the dermatology clinic is warranted due to his previous history of metastatic melanoma, multiple risk factors for metastasis, as well as the poorly understood prognosis of BCC with intravascular invasion.
